# Supplementary material for: Hybrid Models and Biological Model Reduction with PyDSTool
Source: PLoS Comput Biol. 2012 Aug 9;8(8):e1002628. doi: 10.1371/journal.pcbi.1002628 (PMC3415397; doi:10.1371/journal.pcbi.1002628)
Supplement: Text S4 — Complete source code for the PyDSTool package (version 0.88.120504). Includes API documentation and help files linking to web pages. This file is identical to the current public release on Sourceforge.net. (ZIP) [file pcbi.1002628.s004.zip › PyDSTool/html/PyDSTool.Toolbox.optimizers.step.conjugate_gradient_step-pysrc.html]

xml version="1.0" encoding="ascii"?


PyDSTool.Toolbox.optimizers.step.conjugate\_gradient\_step


| Home | Trees | Indices | Help | PyDSTool |
| --- | --- | --- | --- | --- |

Package PyDSTool ::
Package Toolbox ::
Package optimizers ::
Package step ::
Module conjugate\_gradient\_step

[hide private]

[frames] | no frames]

# Source Code for Module PyDSTool.Toolbox.optimizers.step.conjugate\_gradient\_step

```
  1   
  2  # Matthieu Brucher 
  3  # Last Change : 2007-08-29 15:05 
  4   
  5  """ 
  6  Computes the conjugate gradient steps for a specific function at a specific point 
  7  """ 
  8   
  9  import numpy 
 10   


11 -class ConjugateGradientStep(object):


12    """ 
 13    The basic conjugate gradient step 
 14    """ 


15 -  def __init__(self, coeff_function):


16      """ 
 17      Initialization of the gradient step 
 18        - coeff_function is the function that will compute the appropriate coefficient 
 19      """ 
 20      self.oldStep = None 
 21      self.oldGradient = None 
 22      self.coeff_function = coeff_function

 23   


24 -  def __call__(self, function, point, state):


25      """ 
 26      Computes a gradient step based on a function and a point 
 27      """ 
 28      newGradient = function.gradient(point) 
 29   
 30      if 'direction' in state: 
 31        oldGradient = state['gradient'] 
 32        oldStep = state['direction'] 
 33        if all(newGradient == oldGradient): 
 34          coeff = 0 
 35          step = - newGradient 
 36        else: 
 37          coeff = self.coeff_function(newGradient, oldGradient, oldStep) 
 38          step = - newGradient + coeff * state['direction'] 
 39      else: 
 40        coeff = 0 
 41        step = - newGradient 
 42      self.oldGradient = newGradient 
 43      state['gradient'] = newGradient 
 44      state['conjugate_coefficient'] = coeff 
 45      state['direction'] = step 
 46      return step

 47   


48 -def CWConjugateGradientStep():


49    """ 
 50    The Crowder-Wolfe or Hestenes-Stiefel conjugate gradient step 
 51    """ 
 52    def function(newGradient, oldGradient, oldStep): 
 53      return numpy.dot(newGradient.T, (newGradient - oldGradient)) / numpy.dot(oldStep.T, (newGradient - oldGradient))

 54    return ConjugateGradientStep(function) 
 55   


56 -def DConjugateGradientStep():


57    """ 
 58    The Dixon conjugate gradient step 
 59    """ 
 60    def function(newGradient, oldGradient, oldStep): 
 61      return - numpy.dot(newGradient.T, newGradient) / numpy.dot(oldStep.T, oldGradient)

 62    return ConjugateGradientStep(function) 
 63   


64 -def DYConjugateGradientStep():


65    """ 
 66    The Dai Yan conjugate gradient step 
 67    Has good convergence capabilities (same as the FR-PRP gradient) 
 68    """ 
 69    def function(newGradient, oldGradient, oldStep): 
 70      return numpy.dot(newGradient.T, newGradient) / numpy.dot(oldStep.T, (newGradient - oldGradient))

 71    return ConjugateGradientStep(function) 
 72   


73 -def FRConjugateGradientStep():


74    """ 
 75    The Fletcher Reeves conjugate gradient step 
 76    Needs an exact line search for convergence or the strong Wolfe-Powell rules for an inexact line search 
 77    """ 
 78    def function(newGradient, oldGradient, oldStep): 
 79      return numpy.dot(newGradient.T, newGradient) / numpy.dot(oldGradient.T, oldGradient)

 80    return ConjugateGradientStep(function) 
 81   


82 -def PRPConjugateGradientStep():


83    """ 
 84    The Polak-Ribiere-Polyak conjugate gradient step 
 85    Can restart automatically, but needs an exact line search with a uniformely convex function to globally converge 
 86    """ 
 87    def function(newGradient, oldGradient, oldStep): 
 88      return numpy.dot(newGradient.T, (newGradient - oldGradient)) / numpy.dot(oldGradient.T, oldGradient)

 89    return ConjugateGradientStep(function) 
 90   


91 -def FRPRPConjugateGradientStep():


92    """ 
 93    The Fletcher-Reeves modified Polak-Ribiere-Polyak conjugate gradient step 
 94    Can restart automatically and has the advantages of the PRP gradient and of the FR gradient 
 95    """ 
 96    def function(newGradient, oldGradient, oldStep): 
 97      beta = numpy.dot(newGradient.T, (newGradient - oldGradient)) / numpy.dot(oldGradient.T, oldGradient) 
 98      betafr = numpy.dot(newGradient.T, newGradient) / numpy.dot(oldGradient.T, oldGradient) 
 99      if beta < -betafr: 
100        beta = -betafr 
101      elif beta > betafr: 
102        beta = betafr 
103      return beta

104    return ConjugateGradientStep(function) 
105   


106 -def HZConjugateGradientStep():


107    """ 
108    The Hager-Zhang conjugate gradient step 
109    Has good convergence capabilities (same as the FR-PRP gradient) 
110    """ 
111    def function(newGradient, oldGradient, oldStep): 
112      yk = newGradient - oldGradient 
113      beta = numpy.dot((yk - 2*numpy.linalg.norm(yk)/numpy.dot(yk.T, oldStep) * oldStep).T, newGradient) / numpy.dot(yk.T, oldStep) 
114      return beta

115    return ConjugateGradientStep(function) 
116
```

  


| Home | Trees | Indices | Help | PyDSTool |
| --- | --- | --- | --- | --- |

|  |  |
| --- | --- |
| Generated by Epydoc 3.0.1 on Fri May 4 15:24:18 2012 | http://epydoc.sourceforge.net |
